# Investigating the effect of drought stress on expression of *WRKY1* and *EREBP1* genes and antioxidant enzyme activities in lemon balm (*Melissa Officinalis* L.)

**DOI:** 10.1007/s13205-016-0416-8

**Published:** 2016-04-08

**Authors:** Mehdi Rahimi, Mojtaba Kordrostami, Mahmood Maleki, Mohsen ModaresKia

**Affiliations:** 1 Department of Biotechnology, Institute of Science and High Technology and Environmental Sciences, Graduate University of Advanced Technology, End of Haft Bagh-e-Alavi Highway Knowledge Paradise, P.O. Box 76315-117, Kerman, 7631133131 Iran; 2 Department of Biotechnology, Faculty of Agricultural Sciences, University of Guilan, P.O. Box 41635-1314, Rasht, Iran

**Keywords:** Peroxidase, Polyethylene glycol (PEG), Superoxide dismutase, Gene expression

## Abstract

Drought stress is a severe environmental constraint to plant productivity. In this study, gene expression of *WRKY1* and *EREBP1* genes and activity of two enzymes were investigated in two genotypes, G9 (as resistance) and G12 (as susceptible) of 10 days-old lemon balm seedlings. Experiments were done according to factorial design on the base of completely randomized design with three replications for expression analysis and enzyme assays. Seedlings were cultured in MS medium suspensions, including 0, 3, 6, 12 and 15 % w/v PEG 6000. Leaf samples were subsequently collected at 0, 3, 6, 24, 48, 72 h after culture. According to the results of the enzyme assay, SOD activity in resistant genotype was more than in sensitive one. POD activity was high in G9 in severe drought condition and in G12 this activity was increased at initial times of drought stress. The survey results revealed that the expression levels of both genes, *EREBP1* and *WRKY1*, in G9 variety were more than G12, and in this respect we can say that the G9 variety is more resistant to drought.

## Introduction

One of the medicinal herbs is lemon balm (*Melissa officinalis*) which is a perennial herbaceous herb. The leaves, which have a mild lemon aroma, are used to make medicine (Moradkhani et al. 2010). Lemon balm is used alone or as part of various multi-herb combination products. It is used for digestive problems, including upset stomach, bloating, intestinal gas (flatulence), vomiting, and colic; for pain, including menstrual cramps, headache and toothache; and for mental disorders, including hysteria and melancholia (Moradkhani et al. 2010). Environmental stresses, e.g. salinity (of soil and water) and water deficit, are some of the main obstacles in the production of agricultural and horticultural crops in many parts of the world, especially in arid and semi-arid regions like Iran (Moradkhani et al. 2010).

Plants are constantly exposed to environmental changes; if these changes are strong and rapid, they will be considered as stress (Ciarmiello et al. 2011).

Plants have physiological and biochemical strategies for coping with adverse conditions and produce several compounds in response to stress, which need to be investigated and fully understood (Yang et al. 2010). One of the factors which is activated when plants deal with stress, are transcription factors. In fact, transcription factors are proteins (trans-acting) which strengthen or inhibit the expression of genes by binding to specific sequences of DNA, located in the promoter region of target genes. (Riechmann et al. 2000). It is also clear that many families of these factors are influenced by stress including: *DREB* (Novillo et al. 2004; Sakuma et al. 2006), *NAC* *POBox* (Tran et al. 2004; Xue et al. 2006), *WRKY* (Mangelsen et al. 2008), *MYB* (Abe et al. 2003; Yamaguchi-Shinozaki and Shinozaki 2005) and *bZIP* (Kobayashi et al. 2008).

Ethylene-responsive element-binding proteins (*EREBPs*) are members of a family of plant transcription factors. Conserved *EREBP* domains of these proteins bind to the GCC box, an ethylene-responsive promoter element found in many pathogenesis-related (PR) genes (Mizoi et al. 2012). The analyses revealed that *GmEREBP1* is expressed in a root-preferential manner and that *GmEREBP1* mRNA abundance is changed after *H. glycines* infection. *GmEREBP1* mRNA abundance decreased in infected (susceptible) ‘Corsoy 79’ roots, whereas it increased in abundance in infected (resistant) ‘Hartwig’ roots. Furthermore, instead of wounding, ethephon treatment repressed *GmEREBP1* mRNA accumulation in both cultivars. These changes in mRNA steady-state levels suggest that *GmEREBP1* plays a role in soybean *H. glycines* interactions (Mazarei et al. 2002).

WRKY transcription factors are one of the largest families of transcriptional regulators in plants and form integral parts of signaling webs that modulate many plant processes (Sun et al. 2003). New findings illustrate that WRKY proteins often act as repressors as well as activators, and that members of the family play roles in both the repression and de-repression of important plant processes (Ramamoorthy et al. 2008). Mechanisms of signaling and transcriptional regulation are being dissected, uncovering WRKY protein functions via interactions with a diverse array of protein partners, including MAP kinases, MAP kinase kinases, 14-3-3 proteins, calmodulin, histone deacetylases, resistance proteins and other WRKY transcription factors (Eulgem et al. 2000).

There are still few studies, investigating the expression of genes associated with drought stress in plants, especially lemon balm. Since the fundamental principles of any breeding program are determined by studying the genetic parameters, therefore, the knowledge about manner and effect of the genes is essential for the success of breeding programs (Luo 2010). This study aimed to evaluate the expression levels of two genes *WRKY1* and *EREBP1* in lemon balm. These two genes are Geranial responsive elements which have been extracted from chamomile leaf primordial by Ashida et al. (2002).

## Materials and methods

### Plant material

In this study, a total number of 12 genotypes of lemon balm, which were prepared from National Center for Genetic and Biological Resources, were screened for drought resistance.

### Treatments

The seeds of 12 lemon balm genotypes were surface sterilized by treatment with 70 % ethanol for 5 min, followed by commercial bleach (0.5 % sodium hypochlorite) containing 0.05 % Triton X-100 for 20 min, followed by four washes with sterile distilled water. Seeds were stratified in the dark at 4 °C for 3 days. Then, seeds were sown on half-strength MS medium composed of MS basal salts, 1 % agar, and 1 % Sucrose. The pH was adjusted to 5.7 with potassium hydroxide before autoclaving. Plates were sealed and incubated in a growth chamber at 22 °C under a 16-h-light/8-h-dark photoperiod. Then the 10 days old seedlings were transferred to the media containing 0, 3, 6, 9, 12 and 15 % of PEG. Root length, shoot length, root dry weight, shoot dry weight, total biomass and drought resistance indices were evaluated (data not shown). After primary screening genotypes for drought tolerance, two genotypes, G9 (as resistance) and G12 (as susceptible) were selected for gene expression and enzyme assay. Experiments were done according to the factorial design on the basis of completely randomized design with three replications. Ten day-old seedlings were cultured in MS medium suspensions, including 0, 3, 6, 12 and 15 % w/v PEG 6000. Leaf samples were subsequently collected at 0, 3, 6, 12, 24, 48, 72 h after culture.

### Enzyme assay

For enzyme extraction, the plants were grown on severe drought conditions (PEG 15 %) MS medium. About 0.3 g of leaf samples were homogenized with 5 mL of 50 mM buffer solution (containing 0.7 % of NaH_2_PO_4_.2H_2_O and 1.64 % Na_2_HPO_4_.12H_2_O, pH 7.8) subjected to grinding with an ice-cooled mortar and pestle and finally centrifuged at 5000*g*_n_ for 25 min at 4 °C. The supernatant was collected for the determination of superoxide dismutase (SOD) and guaiacol-dependent peroxidase (POD) activity. Peroxidase (POD) EC number (1.11.1.7) (mmol min^−1^ mg protein^−1^) activity was determined following the method described by Chance and Maehly (1955). The POD activity was assayed by guaiacol oxidation and defined as 0.01 absorbance change min^−1^ mg^−1^ protein. The reaction mixture was prepared by adding 400 µL guaiacol (20 mM), 500 µL H_2_O_2_ (40 mM) and 2 mL phosphate (50 mM) in 100 µL enzyme extract. The change in absorbance at 470 nm of the reaction mixture was observed every 20 s up to 5 min. The POD activity was expressed as mmol min^−1^ mg protein^−1^ (Chance and Maehly 1955). SOD activity was measured according to the method used by Chowdhury and Choudhuri (1985) and Zhang and Kirkham (1994). Superoxide dismutase (EC number 1.15.1.1) (IU min^−1^ mg protein^−1^) activity inhibits the photochemical reduction of nitroblue tetrazolium (NBT) at 560 nm. The monitoring of this inhibition is used to assay SOD activity. The reaction mixture was prepared by taking 50 µL enzyme extract and adding 1 mL NBT (50 µM), 500 µL methionine (13 mM), 1 mL riboflavin (1.3 µM), 950 µL (50 mM) phosphate buffer and 500 µL EDTA (75 mM). This reaction was started by keeping reaction solution under 30 W fluorescent lamp illumination and turning the fluorescent lamp on. The reaction stopped when the lamp turned off 5 min later. The NBT photo reduction produced blue formazane which was used to measure the increase in absorbance at 560 nm. The same reaction mixtures without enzyme extract in the dark were used as blanks. The SOD activity was determined and expressed as µMol/g FW · min.

### Gene expression

RNA was extracted using phenol/SDS method based on IHBT (Ghawana et al. 2011) and phenol/SDS (Kingston 2010) protocols with some modification. *WRKY1* and *EREBP1* primers were designed by aligning *WRKY* and *EREBP1* isoforms from *Matricaria, Arabidopsis*, *Robus*, *Glycine max,* *Nicotiana tobbacum, Populus*, etc. using ClustalW software. A 350 base pair conserved segment was found in agree with the region related to lemon balm. Finally, we designed gene-specific primers capable of amplifying this segment in lemon balm *WRKY* and *EREBP1* cDNA. The sequence of oligonucleotides used for the study was as follows: F: *EREBP1* (AB035270.2) (5′-GGACGATGACGTCATCTCCG-3′), R: *EREBP1* (5′- TAGCCGGATCACGAATCTCC3-3′), F: *WRKY1* (AB035271.2) (5′-AATCTTCAGTCTTTCTTCGC-3′), R: *WRKY1* (5′-ACATCTAAAATAGCACCTGG3-3′) F: 18S rRNA (5′-GTAACCCGTTGAACCCCATT-3′) and R: 18S rRNA (5′-CCATCCAATCGGTAGTAGCG-3′). The qRT-PCR was conducted with SYBR Green I (Sigma-Aldrich, St. Louis, MO) as a fluorogenic intercalating dye on a Bio-RAD Real-Time Detection System. Each 20 μL of reaction solution contained 3.5 mmol L^−1^ MgCl_2_, 1 unit PCR buffer, 0.5 μM of each primer, 10 μM dNTPs, 0.5 unit SYBR Green I, 1 unit Taq polymerase (Fermentas Canada), 0.6 μL DMSO (Sigma-Aldrich), and 1.5 μL of template cDNA. A parallel control containing no templates was run to determine contaminations and formation of primer dimers. An internal control using 18srRNA gene also was included. The conditions and parameters for PCR were as follows: an initial denaturing step at 95 °C for 3 min, followed by 40 cycles of 94 °C for 15 s, 55 °C for 20 s, and 72 °C for 20 s. Melting curve analysis of the amplification products was performed at the end of each PCR reaction to ensure that a single PCR product was detected. The relative gene expression of target genes in comparison to the 18SrRNA reference gene was calculated using the Bio-Rad CFX Manager 3.0 Software of the C1000 Touch thermal cycler-CFX96 Real-time PCR (BIO-RAD, Foster city, California, USA). One-way analysis of variance was conducted using SPSS (version 21.0; IBM Corp., Armonk, NY, USA). Treatment means were separated using Fisher’s least significant difference at 0.05 significant levels.

## Results

Analysis of variance for all measured traits is shown in Table 1. Results showed that the interaction between genotype × time was significant for all the measured traits. It shows the genotypes had different reaction in different hours afters the stress.

**Table 1:** Anova for SOD and POD activity in two genotypes

S.O.V	*df*	MS of trait	
		SOD	POD
Genotype	1	55.16^a^	0.002^a^
Time	6	142.91^a^	0.019^a^
Genotype × time	6	39.99^a^	0.026^a^
Error	14	0.08	0.00007
CV%		3.57	6.75

^a^Significant at 1 % probability levels

### SOD activity

**Fig. 1:**
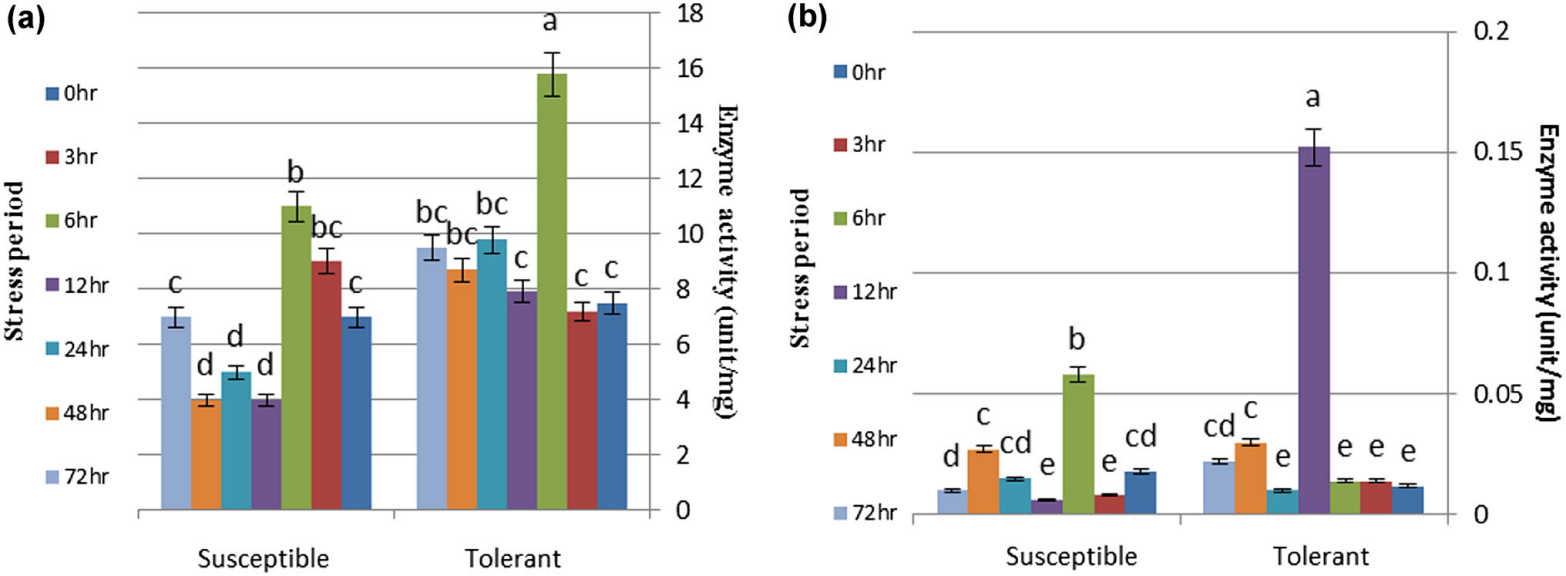
The relative amounts of SOD (**a**) and POD (**b**) enzymes in both resistant and susceptible genotypes. Means with *uncommon* *letters in each column* have significant difference using Duncan test at the 5 % level. *Data points* and *error bars* represent mean and standard deviation from three independent experiments

SOD activity was significantly different in both tolerant and susceptible genotypes under drought stress conditions (Table 1). The maximum SOD activity, in tolerant genotype, was observed 6 h after stress and the minimum was observed after 3 h (Fig. 1a). According to the Fig. 1, it can be concluded that the amount of superoxide dismutase followed a constant rhythm in the tolerant genotype; except for 6 h after the stress, when the amount of the enzyme reached its maximum. The situation was different in the sensitive genotype. The amount of enzyme was increasing from 0 to 6 h after the stress and reached its maximum value at 6 h after stress. At the other times, a constant rhythm was observed. It is true that the amount of SOD reached its maximum at 6 h after the stress in both tolerant and sensitive genotypes, but this amount in tolerant genotype was much more than in the sensitive one.

### Guaiacol-dependent peroxidase activity

Although the patterns of response to drought stress in two genotypes were similar for peroxidase enzyme activity, the amount and intensity of activity for this enzyme were significantly different in both genotypes (Table 1). The maximum POD activity, in tolerant genotype, was observed 12 h after stress (Fig. 1b).

According to the Fig. 1, it can be concluded that the amount of POD followed a constant rhythm in the tolerant genotype; except for 12 h after the stress, when the amount of enzyme reached its maximum. The sensitive genotype had the same situation. Totally, the amount of this enzyme was much higher in the tolerant cultivar compared to susceptible one. In this genotype, the maximum amount of peroxidase was observed 6 h after stress.

### *EREBP1* and *WRKY1* gene expression

**Fig. 2:**
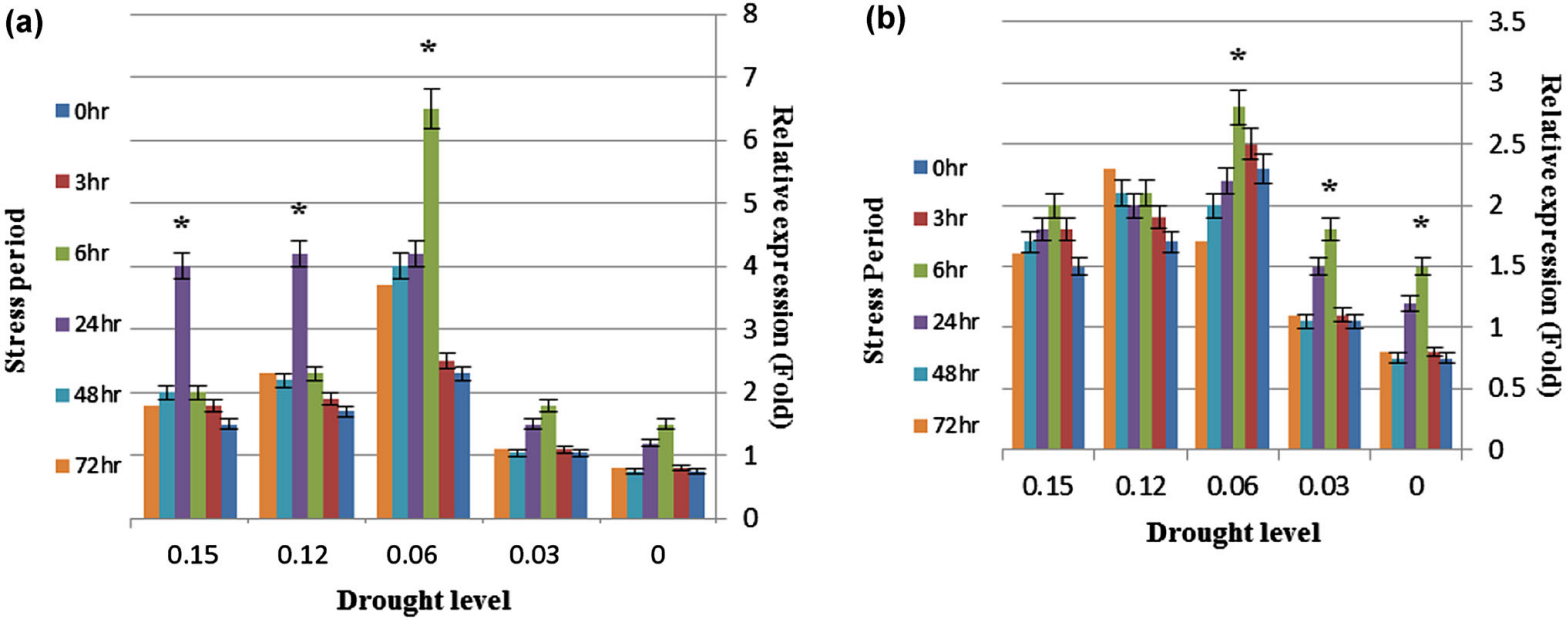
*EREBP1* gene expression level in tolerant (**a**) and susceptible (**b**) genotypes. *Asterisk* showed >3-fold increase in the expression of gene. *Data points* and *error bars* represent mean and standard deviation

According to Fig. 2, it can be seen that *EREBP1* gene expression was increased in tolerant genotype (G9). This Increase was enormous in drought level of 6 %, and 6 h after stress (Fig. 2a). In susceptible genotype (G12), gene expression levels were reduced compared to G9. This reduction was observed 6, 12 and 24 h after stress (in 12 and 6 % PEG) (Fig. 2b). This dramatic reduction was also observed in other levels. In a way the expression level of this gene was very low 24–72 h after stress (Fig. 2b).

**Fig. 3:**
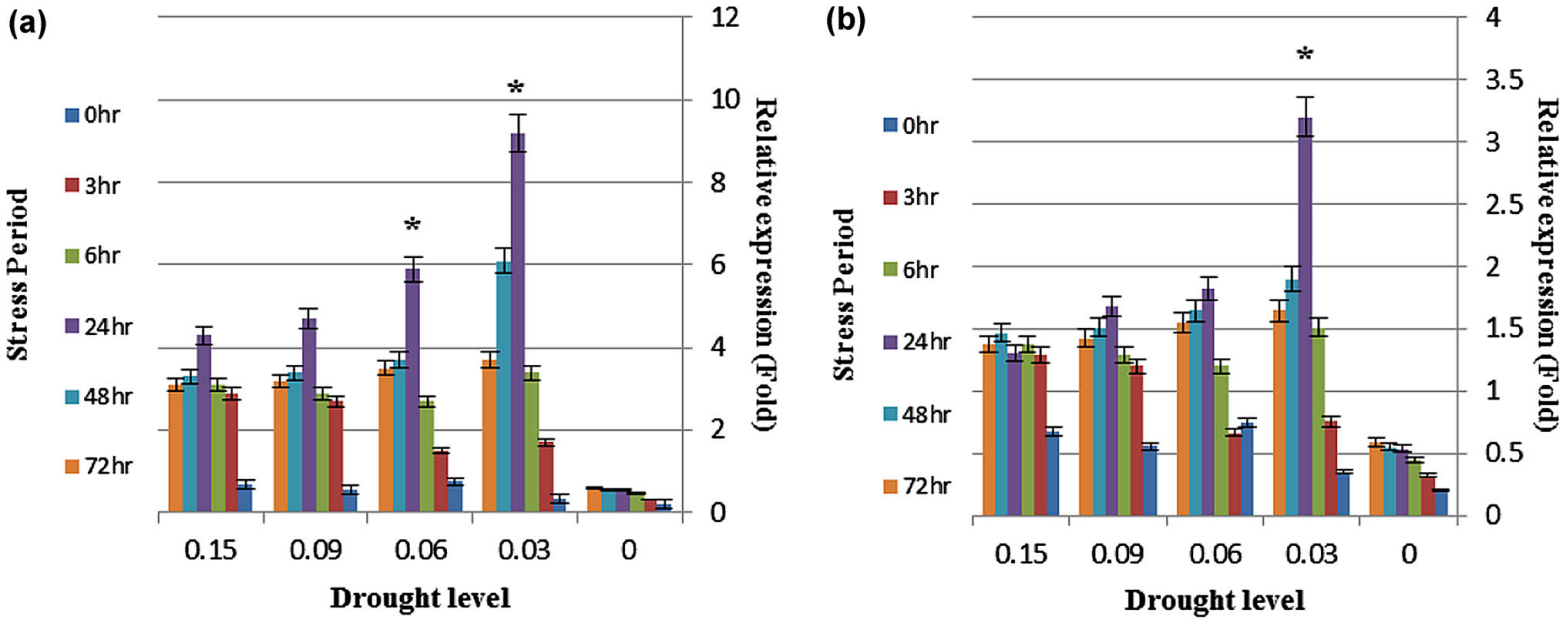
*WRKY1* gene expression level in tolerant (**a**) and susceptible (**b**) genotypes. *Asterisk* showed >3-fold increase in the expression of gene. *Data points* and *error bars* represent mean and standard deviation

*WRKY1* gene expression was increased in tolerant genotype (G9). This increase was enormous at drought level of 3 %, and 24 h after stress (Fig. 3a). Unlike previous gene which was expressed in the early hours of stress, *WRKY1* was expressed in the late hours of stress (Fig. 3a). In susceptible genotype, gene expression levels were reduced compared to tolerant one. This reduction was observed at both 6 and 24 h and 3 and 6 % drought levels (Fig. 3b), in a way that expression level of this gene in G12 was approximately 50 % of G9.

## Discussion

In the susceptible genotype, the maximum SOD activity was observed 6 h after stress and the minimum was observed 48 h after stress (Fig. 1a). In the tolerant genotype, SOD activity was much more than the susceptible one in the same conditions 6 h after stress. In an experiment on wheat, Zhang and Kirkham (1994) showed that SOD activity increased at the beginning of drought stress, but with long periods of drought, the activity of this enzyme was reduced. It appears that increasing the activity of superoxide dismutase in the early stages of drought stress, protects plants from oxidative damage. Decrease or no change in SOD activity after prolonged drought shows that the SOD is not solely responsible for the act of removing ROS in resistant genotype. Thus, by increasing the activity of SOD in the early stages of drought stress, the cleaning intensity of superoxide ion is increased and the damage to the plant will be decreased. In general, researchers reported increase, decrease or no change in SOD activity in different species under drought stress (Gunes et al. 2008; Tohidi-Moghadam et al. 2009).

In the susceptible genotype, the maximum POD activity was observed 6 h after stress. According to the results it can be concluded that POD enzyme activity in the tolerant genotype was increased under severe drought stress. It appears that POD exerts its protective role in the tolerant genotype under severe stress conditions. Khanna-Chopra and Selote (2007) reported that POD activity in tolerant wheat varieties was higher than in susceptible ones. Mercado et al. (2004) showed that POD activity is associated with the ability of maintaining more water in leaves. Similar to what was observed in SOD, in the susceptible genotype, POD was activated faster than in the tolerant one. Increase in POD enzyme activity is definitely a response of the plant to ROS accumulation. It also should be mentioned that there are several enzymatic and non-enzymatic mechanisms of resistance of plants to drought stress.

RNA was extracted using phenol/SDS method with slight modification. This method was based on phenol without using guanidium salts. This buffer contains phenol, SDS, EDTA and sodium acetate salt. SDS and EDTA are inhibitors of RNase and phenol is a strong protein denaturing agent which inhibits the activity of RNase (Wang and Stegemann 2010). In addition to the performance of each buffer component in this method, the substances available in the buffer led to creation of an environment that prevents the oxidation of phenolic compounds and removes them from the extraction buffer. This raises the possibility that RNA can be free of quinone/protein complexes and therefore the extraction and integration of the RNA plate can be easily done (Ghawana et al. 2011). Overall, based on studies, it can be concluded that compared to other methods, phenol/SDS method is more suitable for medicinal plants. Another advantage of the phenol/SDS compared to other methods is relatively short extraction time (approximately 45 min) and low cost of consumables (Accerbi et al. 2010).

Our research confirms Ashida et al. (2002) results; they showed the role of *EREBP1* in drought stress. Expression of *EREBP1* was also increased in their study. Liu and Zhu (1998) investigated the role of *EREBP1* in plants which were exposed to drought stress. In their study, the expression of this gene was increased in the early hours of exposure of plants to drought stress, which somehow confirms our results. This gene has some noticeable roles in other plants: Its role in expression of genes related to ethylene in tobacco (Ohme-Takagi and Shinshi 1995), expression of genes associated with ethylene and flower development in *Arabidopsis* (Jofuku et al. 1994) and its essential role when plants are exposed to cold stress have already been demonstrated (Stockinger et al. 1997). Our study, demonstrated the role of this gene in exposure to drought stress.

Drought, which is often associated with salinity, is an important abiotic stressing factor that has a significant impact on growth, development, survival and function of plants. Thus, understanding the complex mechanisms of drought and salinity is important for agriculture and breeding programs. It has been shown that several *WRKY* proteins are involved in response to drought and salinity (Golldack et al. 2011). For example, overexpression of *OsWRKY11* under the control of HSP101 promoter leads to increased drought resistance, as a reducing agent of wilting leaves, and increases the longevity of green parts of the plant (Wu et al. 2009). Similarly, increased resistance to drought and salinity in *Arabidopsis* may be attributed to induction of ABA stress-related genes by OsWRK72 (Qiu and Yu 2009; Song et al. 2010). OsWRKY08, when its transcripts are increased by PEG, salt or abscisic acid (ABA), is essential for improved tolerance to drought stress in transgenic *Arabidopsis* via positive regulation of two independent abiotic stress responses genes, ABA, AtCOR47 and AtRD21 (Song et al. 2009). Plants that have overexpression of GmWRKY54, show high tolerance to drought and salinity stress, which is probably through the regulation of STZ/Zat10 transcription factor; overexpression of GmWRKY13 leads to increased sensitivity to salt and mannitol stress (Zhou et al. 2008).

New findings illustrate that WRKY proteins often act as repressors as well as activators, and that members of the family play roles in both the repression and de-repression of important plant processes. Like our study, (Mondini et al. 2011) concluded that this transcription factor plays an important role in tolerance to drought and salinity in wheat. The gene acts as a positive regulator of defense responses against pathogens. According to transcription profile, it can be concluded that G9 was more tolerant to drought.

## Conclusion

In conclusion, the results of this study showed that drought-tolerant varieties had followed almost the similar patterns for SOD and POD enzyme activity. In this genotype, both enzymes were at their maximum level in the early hours of stress. In addition, the results showed that the enzymes activity under drought stress in tolerant cultivar was more than in the susceptible one. Gene expression analysis revealed that *EREBP1* gene expression was increased in tolerant genotype (G9). This increase was enormous at drought level of 6 %, and 6 h after stress. *WRKY1* gene expression was increased in tolerant genotype (G9). This increase was enormous at drought level of 3 %, and 24 h after stress. In addition, the results showed that the gene expression under drought stress in tolerant cultivars was more than in susceptible ones. Given that lemon balm is an important medicinal plant in Iran, and taking into consideration the fact that lack of water and salt are the major limiting factors in Iran, analysis of gene expression during stress and identification of genes related to resistance or tolerance are very important.
